# Evidence of neuroplasticity with robotic hand exoskeleton for post-stroke rehabilitation: a randomized controlled trial

**DOI:** 10.1186/s12984-021-00867-7

**Published:** 2021-05-06

**Authors:** Neha Singh, Megha Saini, Nand Kumar, M. V. Padma Srivastava, Amit Mehndiratta

**Affiliations:** 1 Centre for Biomedical Engineering, Indian Institute of Technology (IIT), New Delhi, India; 2 Department of Psychiatry, All Indian Institute of Medical Sciences (AIIMS), New Delhi, India; 3 Department of Neurology, All India Institute of Medical Sciences (AIIMS), New Delhi, India; 4 Department of Biomedical Engineering, All India Institute of Medical Sciences (AIIMS), New Delhi, India

**Keywords:** Stroke, Neurological rehabilitation, Wrist, Metacarpophalangeal joint, Robotic exoskeleton, Transcranial Magnetic Stimulation, Cortical-excitability

## Abstract

**Background:**

A novel electromechanical robotic-exoskeleton was designed in-house for the rehabilitation of wrist joint and Metacarpophalangeal (MCP) joint.

**Objective:**

The objective was to compare the rehabilitation effectiveness (clinical-scales and neurophysiological-measures) of robotic-therapy training sessions with dose-matched conventional therapy in patients with stroke.

**Methods:**

A pilot prospective parallel randomized controlled study at clinical settings was designed for patients with stroke within 2 years of chronicity. Patients were randomly assigned to receive an intervention of 20 sessions of 45 min each, five days a week for four weeks, in Robotic-therapy Group (RG) (n = 12) and conventional upper-limb rehabilitation in Control-Group (CG) (n = 11). We intended to evaluate the effects of a novel exoskeleton based therapy on the functional rehabilitation outcomes of upper-limb and cortical-excitability in patients with stroke as compared to the conventional-rehabilitation. Clinical-scales– Modified Ashworth Scale, Active Range of Motion, Barthel-Index, Brunnstrom-stage and Fugl-Meyer (FM) scale and neurophysiological measures of cortical-excitability (using Transcranial Magnetic Stimulation) –Motor Evoked Potential and Resting Motor threshold, were acquired pre- and post-therapy.

**Results:**

No side effects were noticed in any of the patients. Both RG and CG showed significant (*p* < 0.05) improvement in all clinical motor-outcomes except Modified Ashworth Scale in CG. RG showed significantly (*p* < 0.05) higher improvement over CG in Modified Ashworth Scale, Active Range of Motion and Fugl-Meyer scale and FM Wrist-/Hand component. An increase in cortical-excitability in ipsilesional-hemisphere was found to be statistically significant (*p* < 0.05) in RG over CG, as indexed by a decrease in Resting Motor Threshold and increase in the amplitude of Motor Evoked Potential. No significant changes were shown by the contralesional-hemisphere. Interhemispheric RMT-asymmetry evidenced significant (*p* < 0.05) changes in RG over CG indicating increased cortical-excitability in ipsilesional-hemisphere along with interhemispheric changes.

**Conclusion:**

Robotic-exoskeleton training showed improvement in motor outcomes and cortical-excitability in patients with stroke. Neurophysiological changes in RG could most likely be a consequence of plastic reorganization and use-dependent plasticity.

*Trial registry number*: ISRCTN95291802

**Supplementary Information:**

The online version contains supplementary material available at 10.1186/s12984-021-00867-7.

## Introduction

Stroke is one of the leading causes of mortality and morbidity worldwide [1]. Flexor hypertonia of the wrist is one of its common presentations. Post-stroke, the ability to actively initiate extension movement at the wrist and fingers is one of the indicators of the motor recovery [2, 3]. Regaining hand function and Activities of daily living (ADL) is particularly impervious to therapy owing to fine motor control needed for the distal-joints [4]. Conventional rehabilitation therapy is time taking, labor-intensive and subjective. Therapists usually have a high clinical load and a lack of evidence-based technologies to support them, resulting in therapist burnout and a healthcare system that cannot provide appropriate or effective rehabilitation services [5].

Although rehabilitation with neuro-rehabilitation robots has shown encouraging clinical results [5–18], it is currently limited to a very few hospitals and not widely used because of the associated high-cost and an infrastructural-requirement to station these large and complex devices with a high set-up time and limited usability [19–21]. Rehabilitation strategies need to take into account the multifaceted nature of the disability, which is self-changing (progressing or improving), i.e. itself changes with time and requires a multimodal approach. Hence, the assistive device needs to be flexible and adaptive enough to accommodate the needs of a large patient population.

An effective rehabilitation device for the upper-limb should be able to facilitate a specific pattern of coordinated movements of joints, especially for a hand. However, this particular coordination is currently not integrated with any of the commercially available devices, where they mostly focus on movements of the specific individual joint in isolation [22]. For a healthy subject, extending the wrist naturally leads to flexion of the fingers. Semi-extension of the wrist with grasped fingers contributes to ADL movements; which is also commonly disrupted in patients with stroke due to flexor hypertonia. These complex movement patterns have been disintegrated during conventional physiotherapy into few simpler tasks, for example, holding a glass of water consists of sub-tasks like grasping the glass with the fingers in the motion of flexion while wrist in 30–40 degree semi-extended position, and releasing it with fingers being in extension and wrist coming back to the neutral position with flexion as elaborated in [23]. Hence, a device that can simulate the movement pattern of wrist extension along with finger flexion (such as in ADL), maintaining inter-joint coordination with the limited number of actuators making the device less complex, is the need of the hour. Only two devices, Hand Mentor and HWARD, allow hand and wrist synchronization [24]. The Hand Mentor Pro rotates wrist with Metacarpophalengeal (MCP) placed at a constant angle with respect to the wrist, but lacks flexion (grasp) and extension (release) of MCP, also it can’t accommodate patient-centric ROM and speed. Though HWARD synchronized wrist and MCP and provided seminal evidence of reorganization of the brain through robotic-therapy sessions, it had few other challenges such as limited range of motion, no patient-centric muscle-specific training, finger opening requirement, manual adjustment of force at pneumatic cylinders and device having ~ 25 kg of weight. Hence, the challenges remained to simplify the complex design and therapy protocols into simple, lightweight, user-friendly devices that are convenient with a potential to use even in home-settings. Moreover, the device must show efficacy for a broader community while being cost-effective for low and middle-income countries with limited research on rehabilitation [25–27]. Our device attempt to address the key limitations which other commercial devices faced.

In our previous work, we have designed a robotic hand exoskeleton for rehabilitation of the wrist and MCP joint, to synchronize wrist extension with finger-flexion and wrist-flexion with finger extension [28]. It is a prototype device with the potential of being simple and easy to operate exoskeleton rehabilitation device for low-resource settings in the future. The exoskeleton targets spasticity through a synergy-based rehabilitation approach while also maintaining patient-initiated therapy through residual muscle activity for maximizing voluntary effort. The lightweight and portable device indicated an improvement in quantitative motor clinical outcomes in patients with chronic stroke [28].

The aim of the present study was twofold. The first objective was to assess the clinical effectiveness of the novel robotic-exoskeleton device [28] and the second is a comparison of its clinical effectiveness with conventional upper-limb rehabilitation. There is a considerable amount of literature documented for neurorehabilitation robots that takes into account the specific, repetitive, and timed movement goals [29] with maximizing voluntary residual muscle activity, real-time visual performance biofeedback, and proprioceptive feedback for sensorimotor integration. These features might give the robotic-therapy a notch over dose-matched conventional therapy [30, 31]. As the exoskeleton device also has these features [28], thus, we hypothesized that exoskeleton-based rehabilitation therapy might also encourage clinically relevant neuroplasticity with expected better clinical outcomes for distal joints in patients with stroke than the dose-matched conventional rehabilitation.

## Materials and methods

More than 300 patients (n > 300) were screened in the out-patient clinic of the Department of Neurology, AIIMS, New-Delhi over three years from July-2016 to January-2019. Stroke diagnosis was established clinically in all the patients. All clinical assessments and standard care were given to the patients with stroke by a trained physiotherapist. Institutional Review Board (IRB) at All India Institute of Medical Sciences (AIIMS), New-Delhi, India, approved the study under protocol-number IEC/NP-99/13.03.2015 and was registered with clinical trial number ISRCTN95291802. All the patients signed the written informed consent before enrolment.

### Study-design

A pilot prospective parallel randomized controlled study at clinical settings was designed which included pre- and post-clinical-outcome measures of therapeutic intervention. Once enrolled, patients were then randomized under two groups- the robotic-therapy group (RG) and the Control-Group (CG). The RG received robotic therapy for 45 min of individual sessions per day for 20 therapy sessions (5 days a week for 4 weeks). The CG received 45 min per day for 20 therapy sessions (5 days a week for 4 weeks) of conventional physiotherapy training. Both the groups continued the care according to the current clinical standards practice in terms of medication as prescribed by the neurologist. The same therapist provided therapy sessions to all patients in both the groups. The person performing the data analysis was blinded to the individual data.

### Patient enrolment

**Fig. 1:**
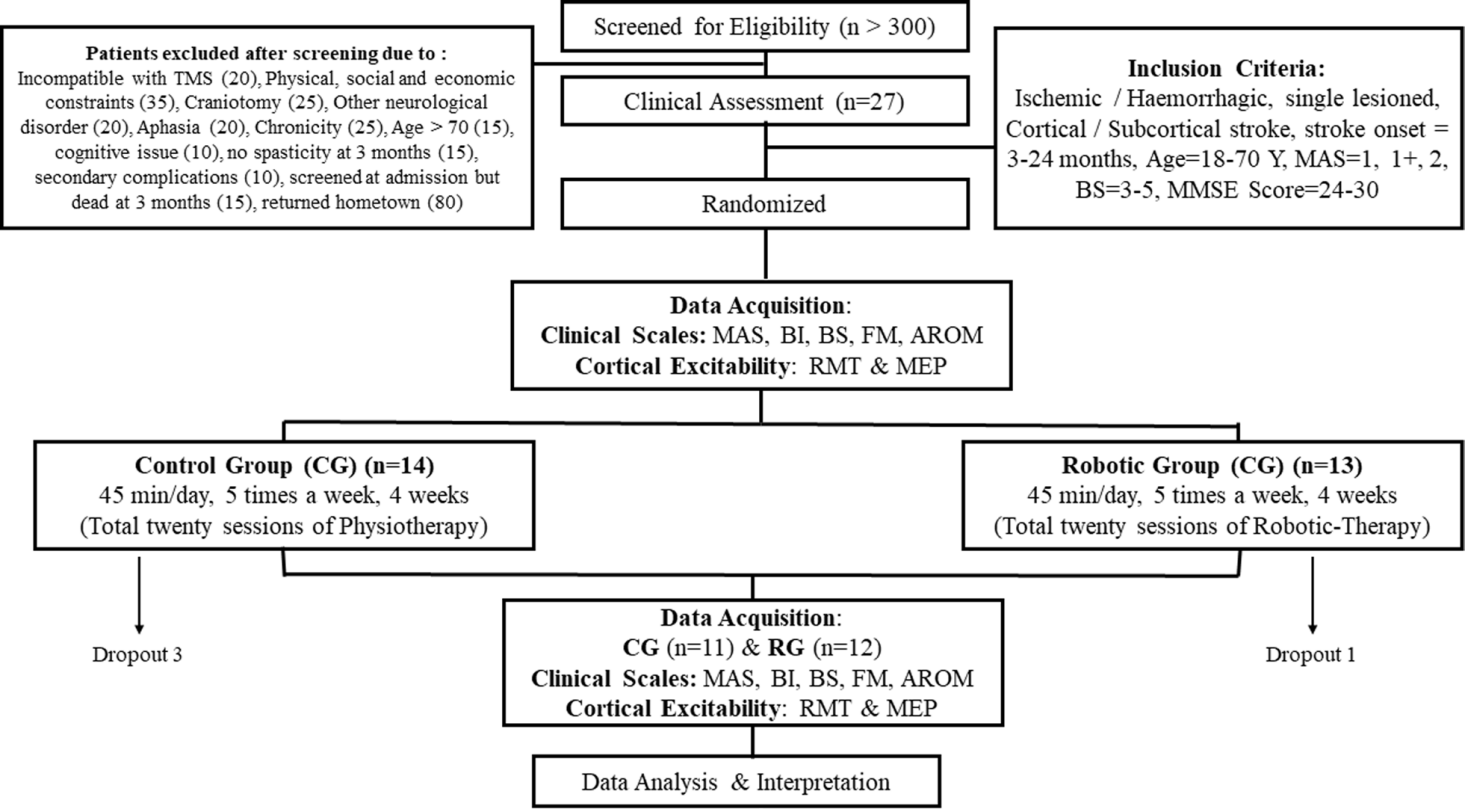
Patient Enrolment Consort

Patients were enrolled based on inclusion-criteria, age 18–70 years, having ischemic / hemorrhagic stroke within 3–24 months, Mini-Mental Scale (MMS) = 24–30; Brunnstrom stage (BS) = 3–5; Modified Ashworth Scale (MAS) = 1, 1 + , 2 (Fig. 1). Patients with contra-indication to Transcranial Magnetic Stimulation (TMS), no detectable Electromyogram (EMG) activity and any other progressive neurological or cognitive disorders were excluded from the study. The enrolled patients were allocated based on a predefined allocation sequence. Simple randomization was performed using opaque envelopes within which color cards signified the groups. Patients were instructed to choose the opaque envelopes in the predefined sequence. The cards they choose signified the group they were enrolled in for the study. Randomization, outcome measurements, and data analysis were performed by a different individual not involved in the intervention.

### Data collection

All the participants underwent clinical assessment; a pre-therapy assessment a day before the randomization process and before initiation of robotic or conventional training. The post-therapy assessment was performed a day after the completion of intervention by a trained physiotherapist with more than 5 years of experience.

#### Clinical scale measures (primary outcomes)

The primary outcomes were: the level of spasticity at wrist joint measured by Modified Ashworth Scale (MAS 0–4), range of voluntary wrist movement defined in terms of Active Range of motion of wrist (AROM 0^0^–70^0^) as measured by a goniometer, stage of stroke recovery measured by Brunnstrom Stages (1–7), Barthel Index (0–100) and functional and sensorimotor-control of upper-limb as measured by Fugl-Meyer Scale Upper Limb (FMU/L 0–66), segregated into its wrist hand component (FMW/H) and shoulder elbow component (FMS/E) (Fig. 1).

#### Cortical-excitability measures using TMS (secondary outcomes)

Patients were allowed to sit comfortably on the chair, kept forearm pronated, elbow-joint at 90–120° flexion, wrist-joint at a neutral position, and fingers at rest. Single-pulse TMS at 100% Motor Threshold was given to all the patients to evoke the Motor Evoked Potential (MEP) signal, using a flat 70 mm figure of eight coils (type D70 (AC), serial no. 0326, Magstim Rapid^2^, Magstim, UK), at the cortical representation of the Extensor Digitorum Communis (EDC) muscle (on the motor-cortex with reference to the EEG cap) of the ipsilesional and contralesional-hemisphere. Cortical-excitability was measured in terms of Resting Motor-Threshold (RMT) and MEP amplitude using TMS over ipsilesional and contralesional-hemisphere according to the standard protocol [32]. RMT was defined as the minimum intensity of TMS required to elicit an MEP in target contralateral-muscle in 5/10 trials, recorded in EMG, over the muscle cortical representation in the primary motor cortex. MEP encapsulates information relevant to the cortical excitability of the brain, conduction and functional integrity of the corticospinal-tract [33]. MEP should be ≥ 50µv peak-to-peak amplitude at the hotspot in 5/10 consecutive trials. Five MEP signals out of 10 consecutive trials were averaged.

### Robotic therapy-sessions

**Fig. 2:**
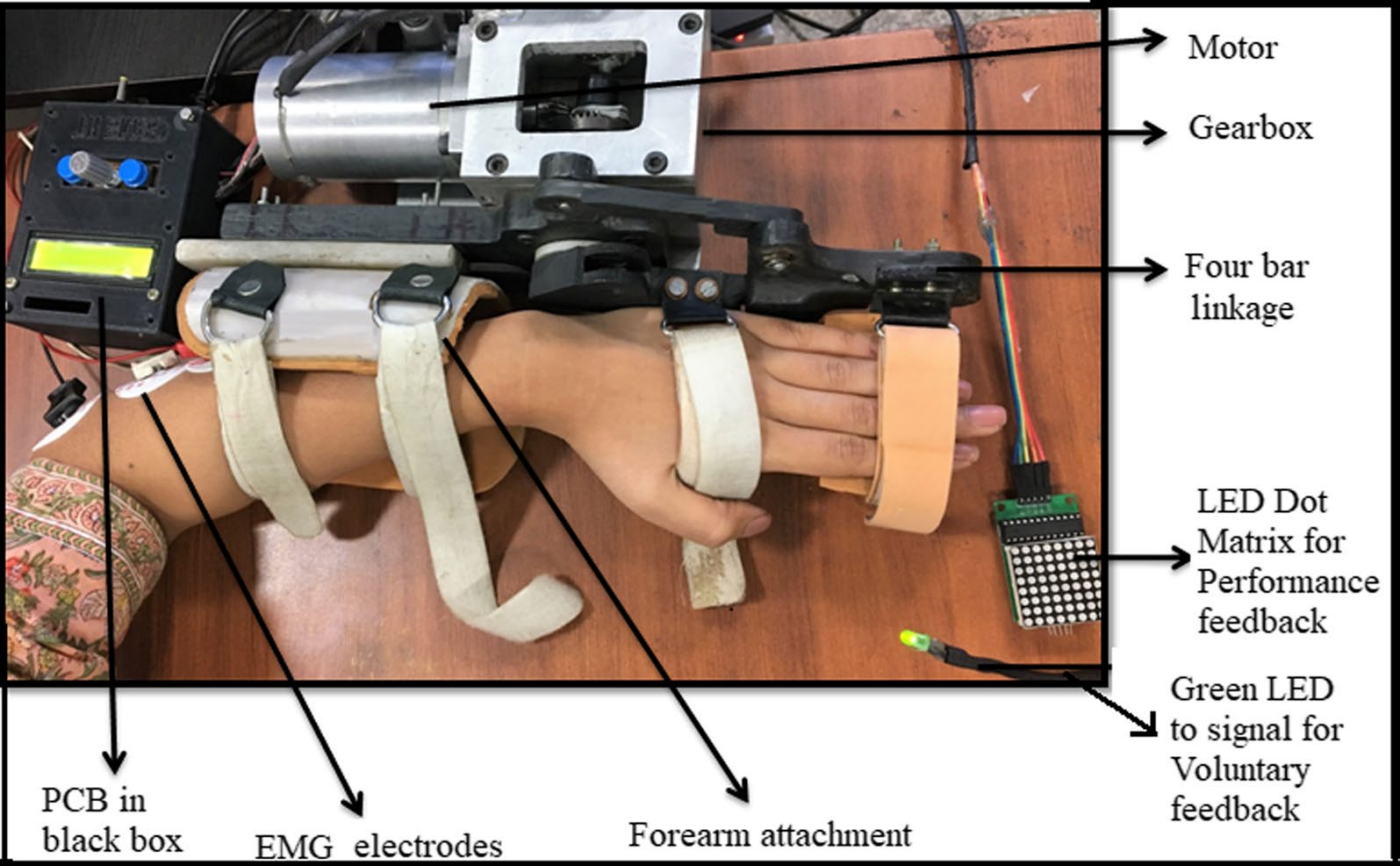
Whole set-up of exoskeleton with performance biofeedback, voluntary cue and PCB in the black control box which also works as user interface [28]

An electromechanical robotic-exoskeleton was developed for rehabilitation of wrist-joint and fingers-joint [28] (Fig. 2 and Additional file 1: Figure S1). Stages of motion sequence were: wrist at the neutral position, finger extension (baseline position) → wrist extension finger flexion (final position) → back to wrist flexion, finger extension (towards baseline position); with a constant speed (28 degrees/second) for all the patients. The device was safe, user-friendly and patient-centric as per the clinical presentation: with customizable motion-parameters: (i) initial-position for a range of motion (ROM), (ii) final-position for ROM, (iii) speed, (iv) residual muscle-activity and (v) height of finger-support. All sessions were given at the hospital set-up under the supervision of an expert clinician.

Each 45 min robotic-therapy session consisted of approximately 250 trials of 10 s each, excluding the set-up time, breaks, donning and doffing of the exoskeleton or consultation which was an additional 10–15 min. Patients were advised to take 5 min break for rest in between the therapy-session if there is a feeling of pain or fatigue, this time was then added to the total therapy time, keeping the active therapy session to 45 min consistently.

Patient hands were stabilized in the exoskeleton device with the velcro straps in the neutral position and therapy required to extend the wrist in a neutral position only (with no ulnar/radial deviation). The device is actively initiated by Electromyogram (EMG) activity of EDC muscle [28, 34–37] with robot motion-triggered only if the EMG thresholds (set with the consensus of the therapist at the time of first therapy sitting) are crossed and it provides an interactive adaptive performance visual biofeedback in real-time [28]. At baseline position, the patient tries to extend the wrist voluntarily for the first three seconds after the green LED cue. If the EMG crosses the predefined threshold, the exoskeleton will be triggered for an assisted wrist extension and finger flexion movement. Once it reaches the final position, the exoskeleton then assists the patient’s hand back to the baseline position, wrist flexion with finger extension. Simultaneous with this motion assistance, the performance feedback is given to the patient in real-time. To ensure each cycle lasted 10 s, a delay of a few sec/milliseconds (depending on the individual patient’s completion time) was provided after the completion of each cycle [28]. If the EMG threshold is not crossed, the exoskeleton will not assist the movement and the trial cycle is reset to begin again for the patient to try harder with the repeated three seconds voluntary cue. The configurability of the threshold was adjusted during the study manually and individually using the BIOPAC MP150 EMG acquisition software according to the residual EMG activity of an individual patient with the advantage of making the system patient-specific and including patients with minimal residual muscle activity in the protocol. At pre-therapy, the amplitude of the threshold was in the range of 0.101 ± 41.74 V (amplified with gain = 2000, Band Pass Filter = 10-500 Hz, Notch Filter = 50 Hz, Sampling Frequency = 1000 Hz) for our patient cohort. The final range of motion was incremented during the intervention according to the comfort of the patient. For further details on the device, please refer to our previous work Singh et al. [28].

### Conventional therapy-sessions

The conventional therapy session was conducted for 45 min per day for 5 days a week for 4 weeks. The type of activity, intensity and frequency was based on the baseline clinical presentation of the patient as reflected by clinical scales (MAS, FMA, BI, BS, and Range of motion). More details on the conventional-therapy-session and protocol are presented in the Additional file 1.

### Data analysis

Data analysis was performed in MATLAB R2018a (MATHWORKS®). The data were tested for normality using the Shapiro–Wilk test and was found that clinical measures were not normally distributed in CG. Hence, non-parametric Wilcoxon signed-rank were used for intragroup-comparison of differences in post–pre-therapy within the group, and non-parametric Mann–Whitney tests were used for intergroup-comparison of RG and CG. Interhemispheric-asymmetry for pre and post-therapy measures were calculated and was tested using Wilcoxon signed-rank test. Two-way repeated measure ANOVA was applied to assess the effect of time (two levels-pre and post) and side (two levels-ipsilesional and contralesional) on RMT. Regression and correlation analyses were performed to investigate the relationship of recovery parameters-TMS neurophysiological parameter with the clinical outcome scales. A p-value < 0.05 was considered statistically significant and a Bonferroni correction was applied for post-hoc outcome measure tests. MAS score of 1, 1 + , 2, 3, 4 was mapped as 1, 1.5, 2, 3, 4 for all statistical calculation purposes, respectively, same as suggested by Rong et al. [13]. The change in the parameter values are expressed in terms of percentages as the ratio of the difference between post-therapy and pre-therapy scales normalized to their pre-therapy scales (in discussion section for easy interpretation).

## Results

Twenty-seven patients who met the eligibility criteria were randomized and allocated into two groups- RG (n = 13) and CG (n = 14). One patient (n = 1) in RG and three patients (n = 3) in CG could not complete the therapy, thus, the data were excluded from further analysis. All patients in RG (n = 12) and CG (n = 11) (all right-handed patients with stroke, age = 41.9 ± 11.1 years, Male:Female = 19:4) completed successfully the therapy-sessions in 30–34 days, Table 1 represents the demographic details of all the patients. The CG (n = 11) included patients with stroke, lesion locations with sub-cortical in five (n = 5) and cortical in six (n = 6) patients. RG (n = 12) included patients with stroke, lesion locations with sub-cortical in six (n = 6) and cortical in six (n = 6) patients. The volume of the lesion was 15.98 ± 23.6 cm^3^ in CG and 25.37 ± 45.48 cm^3^ in RG. There were no significant (*p* > 0.05) differences in the pre-therapy measures in terms of clinical scales and lesion volume among both the intervention groups (Table 1). At pre-therapy measurements, MEP was evoked only for 9 patients (RG = 4, CG = 5) out of a total of 23 in ipsilesional-hemisphere, and for all patients in contralesional-hemisphere. The thresholds for triggering of the exoskeleton changed from 0.101 ± 41.74 V at day 1 to 0.383 ± 171 V at day 20 for our RG patients' cohort with a relative increase by ~ 3 times. No side effects or adverse effects were noticed in any of the patients during the study.

**Table 1:** Details of patients with stroke enrolled in Robotic Group and Control Group

Measures	Pre-therapy measures robotic group (n = 12) Mean ± SD	Pre-therapy measures control group (n = 11) Mean ± SD	*p*-value
Age (years)	41.1 ± 12.8	42.7 ± 9.3	0.75
Chronicity (months)	13.8 ± 9.1	10.3 ± 5.0	0.47
MAS	1.75 ± 0.2	1.86 ± 0.5	0.46
AROM (degrees)	15.0 ± 9.7	13.6 ± 7.7	0.34
BI	74.1 ± 12.4	69.5 ± 12.9	0.41
BS	3.67 ± 0.7	3.72 ± 1	0.9
FMU/L	36 ± 7.7	37.45 ± 9.1	0.98
FMW/H	9.7 ± 2.7	11.45 ± 2.9	0.27
FMS/E	26.2 ± 5.6	26 ± 7.07	0.78
Lesion Volume (cm^3^)	25.3 ± 45.48	15.9 ± 23.6	0.97

*MAS (max 4)* Modified Ashworth Scale, *AROM (max 70)* Active Range of Motion

*BI (max 100)* Barthel Index, *BS (max 7)* Brunstorm Stage

*FMU/L (max 66)* Fugl-Meyer Upper Limb *Scale, FMW/H (max 24)* Fugl-Meyer Wrist Hand

*FMS/E (max 42)* Fugl-Meyer Shoulder Elbow

### Comparison of clinical-scales

Post-therapy, all clinical scales in both groups did show significant changes in improvement, except for MAS in CG. However, all clinical scales (MAS, AROM, FM, and FMW/H) in RG showed improvement with statistically significant changes over CG. MAS in RG changed from 1.75 ± 0.2 to 1.29 ± 0.3 and in CG from 1.86 ± 0.5 to 1.59 ± 0.6 showing a significant decrease in spasticity at wrist-joint in RG and not in CG (RG-p = 0.0009, CG-p = 0.12) with significant (p = 0.03) intergroup changes (Table 2). AROM and BI, in both groups, showed statistically significant differences. AROM significantly increased in both the groups, from 15^0^ ± 9.7^0^ to 34.6^0^ ± 14.5^0^ in RG (*p* = 0.0004) and from 13.6 ± 7.7^0^ to 20.0^0^ ± 8.1^0^ in CG (*p* = 0.002). However, RG manifested statistically significant AROM scores as compared to CG as intergroup-comparison did evidence significant differences (*p* = 0.02) (Table 2). BI changed from 74.1 ± 12.4 to 89.1 ± 7.9 in RG (*p* = 0.0009) and from 69.5 ± 12.9 to 82.7 ± 14.3 in CG (*p* = 0.0009); the intergroup comparison did not show any significant differences (p = 0.82). BS showed statistically significant differences in both groups, RG increased from 3.6 ± 0.7 to 4.8 ± 0.9 (*p* = 0.0004) and CG  increased from 3.7 ± 1 to 4.4 ± 1.2 (*p* = 0.015). The intergroup comparison did not show significant differences between both groups (*p* = 0.311) (Table 2).

**Table 2:** Comparison of clinical-scales, cortical-excitability (in ipsilesional and contralesional-hemisphere) and interhemispheric parameters in Robotic Group with Control Group

Groups	Robotic-therapy group				Control group				Intergroup *p*-value	Bonferroni correction
Outcomes	Pre-Therapy	Post-Therapy	Difference of mean	Robotic group *p*-value	Pre-therapy	Post-therapy	Difference of mean	Control group *p*-value		
	Mean + Std dev				Mean + Std dev					
Clinical scales										
MAS	1.75 ± 0.2	1.29 ± 0.3	0.46	0.0009	1.86 ± 0.5	1.59 ± 0.6	0.27	0.12	0.03*	0.19
AROM	15.0^0^ ± 9.7	34.5^0^ ± 14.5^0^	19.58^0^	0.0004	13.6^0^ ± 7.7^0^	20.0^0^ ± 8.0^0^	6.4	0.002	0.02*	0.07
BI	74.1 ± 12.4	89.1 ± 7.9	15	0.0009	69.5 ± 12.9	82.7 ± 14.3	13.18	0.0009	0.82	0.48
BS	3.6 ± 0.7	4.8 ± 0.9	1.16	0.0004	3.72 ± 1	4.4 ± 1.2	0.73	0.015	0.311	0.27
FMU/L	36 ± 7.7	50.2 ± 6.5	14.2	0.0004	37.4 ± 9.1	45.4 ± 9.7	8	0.0009	0.039*	0.04^#^
FMW/H	9.7 ± 2.7	16.6 ± 4.3	6.9	0.0004	11.4 ± 2.9	15.1 ± 3.6	3.73	0.0009	0.012*	0.01^#^
FMS/E	26.2 ± 5.6	33.5 ± 3.8	7.3	0.0009	26 ± 7.07	29.8 ± 7.08	3.8	0.002	0.13	0.28
∆FM W/H	0.73 ± 0.45				0.33 ± 0.14				0.012*	0.01^#^
Cortical-excitability										
RMT IL	95.3 ± 7.8	79.58 ± 14.38	15.17	0.0039	89 ± 16.03	85.18 ± 17.9	3.82	0.12	0.027*	0.02^#^
MEP A IL	39.4 ± 60.4	94.3 ± 63.2	54.9	0.048	38.1 ± 55.9	38.24 ± 40	0.14	0.312	0.014*	0.04^#^
RMT CL	67.33 ± 10.1	65.08 ± 11.12	2.25	0.051	68.09 ± 11.7	66.18 ± 12.57	1.91	0.052	0.87	0.65
MEP A CL	506.33 ± 247	355.3 ± 191.5	151.03	0.23	200.2 ± 77	185.4 ± 268.3	14.8	0.41	0.65	0.37
RMT_asym_	1.43 ± 0.21	1.25 ± 0.31	0.18	0.012	1.33 ± 0.32	1.30 ± 0.28	0.03	0.59	0.028*	0.035^#^
∆RMT_ipsi_	0.16 ± 0.12				0.04 ± 0.09				0.023*	0.017^#^
∆RMT _asym ratio_	0.12 ± 0.14				0.011 ± 0.1				0.028*	0.034^#^

*Shows the statistical significance differences (*p* < 0.05) between RG and CG, ^#^Shows the statistical significance with bonferroni correction applied

*MAS (max 4)* Modified Ashworth Scale, *AROM (max 70)* Active Range of Motion

*BI (max 100)* Barthel Index, *BS (max 7)* Brunstorm Stage

*FMU/L (max 66)* Fugl-Meyer Upper Limb *Scale, FMW/H (max 24)* Fugl-Meyer Wrist Hand

*FMS/E (max 42)* Fugl-Meyer Shoulder Elbow, MEP *A( µv)* MEP Amplitude, RMT (%)

*IL* Ipsilesional, *CL* Contralesional, *RMT*_*asymm*_  = (RMT Ipsilesional / RMT contralesional), *∆RMT*_*asymm-ratio*_ = (Post RMT_asymm_—Pre RMT_asymm_)/Pre *RMT*_*asymm*_   = Relative improvement in RMT ratio

*∆RMT*_*ipsi*_ = (Pre RMT _Ipsi_–Post RMT _Ipsi_)/*Pre RMT *_*Ipsi*_ (RMT decreases in case of improvement) = Relative decrease/improvement in ipsilesional RMT

*∆FMW/H* = (Post FMW/H–Pre FMW/H)/ Pre FMW/H = Relative improvement in Fugl-Meyer (W/H)

FMU/L scores measure sensorimotor-control gain of upper-limb. FMU/L for RG increased from 36 ± 7.7 to 50.2 ± 6.5 (*p* = 0.0004) and from 37.4 ± 9.1 to 45.4 ± 9.7 for CG (*p* = 0.0009) (Additional file 1: Figure S2). RG showed improvement in sensorimotor scores as compared to CG with significant (with bonferroni corrected *p* = 0.04) differences in intergroup comparison (Table 2). For the proximal part-Shoulder/Elbow component (FMS/E), both groups showed a statistically significant increase, RG changing from 26.2 ± 5.6 to 33.5 ± 3.8 (*p* = 0.0009) and from 26 ± 7.07 to 29.8 ± 7.08 in CG (*p* = 0.002). However, the intergroup comparison did not show any significant (*p* = 0.13) differences. For the distal-part FMW/H, both groups showed a statistically significant increase, in RG increasing from 9.7 ± 2.7 to 16.6 ± 4.3 (p = 0.0004) and in CG increasing from 11.45 ± 2.9 to 15.18 ± 3.6 (*p* = 0.0009). RG manifested statistically significant (with bonferroni corrected *p* = 0.01) sensorimotor improvement in intergroup comparison over CG (Table 2).

### Comparison of cortical-excitability

#### Ipsilesional-hemisphere

MEP, in some patients with stroke, was not recordable even after delivering TMS-stimuli at the highest possible intensity, possibly due to decreased cortical-excitability in stroke as also reported by [38–42]. In those patients with no MEP recorded, RMT is taken as a value of 100, as has been suggested in the literature [43, 44]. Change in RMT showed statistically significant differences in post-therapy in RG as compared to CG. Post-therapy, RG showed a significant decrease in RMT from 95.3 ± 7.87 to 79.5 ± 14.3 (*p* = 0.0039), whereas CG showed a decrease from 89 ± 16.03 to 85.1 ± 17.9 (*p* = 0.12). RG manifested statistically significant improvement in RMT with bonferroni correction applied (*p* = 0.02) (Table 2). RG also evidenced a significant increase in MEP amplitude from 39.4 ± 60.4 µv to 94.3 ± 63.2 µv (*p* = 0.048) and in CG, MEP almost remained the same (~ 38 µv) pre-to-post-therapy (*p* = 0.312). RG manifested statistically significant improvement in MEP amplitude with bonferroni correction applied (p = 0.04). A decrease in RMT (*p* = 0.02) and an increase in MEP amplitude (*p* = 0.0142) were seen in RG as compared to CG (Table 2).

In this study, ~ 54% of patients in CG and 67% of patients in RG did not evoke MEP at the pre-therapy measurements. In CG, MEP was evoked only in 5/11 patients at the pre-therapy measurements and was observed in 6/11 patients at post-therapy. However, it is worth noting that in RG, measurable MEP was evoked only in 4/12 patients at the pre-therapy measurements, and post-therapy MEP was observed in 9/12 patients, thus, 5 additional patients showing MEP after robotic therapy intervention. These five additional patients having no MEP amplitude at 100% stimulation intensity in pre-therapy, were observed to have MEP amplitude of 136.6 ± 38.4 µv in post-therapy at 73.0 ± 9.64% stimulation intensity. For these five patients, an increase in clinical scales was also observed as; FMW/H increase from 8.2 ± 2.4 to 16.0 ± 4.1 (an absolute increase of 7.8 ± 2.3), BI from 70.0 ± 11.7 to 92.0 ± 8.3 (an absolute increase of 22 ± 11.7) and AROM from 15.0^0^ ± 5.0^0^ to 37.0^0^ ± 2.7^0^ (an absolute increase of 22^0^ ± 2.7^0^) in RG.

#### Contralesional-hemisphere

There were no significant changes shown by the contralesional-hemisphere. Both RG and CG evidenced minimal differences in RMT (~ 2% in both groups) (Table 2). RG showed decrease from 67.3 ± 10.07 to 65.08 ± 11.1 (p = 0.051) and CG from 68.09 ± 11.7 to 66.1 ± 12.5 (*p* = 0.052). Intergroup comparison too did not show statistically significant differences (*p* = 0.87) (Table 2). MEP amplitude in RG decreased from 506.3 ± 247 to 355.3 ± 191.5 µv (*p* = 0.23) and from 200.2 ± 77 µv to 185.4 ± 268.3 µv in CG (*p* = 0.41). MEP amplitude observed a considerably higher decrease (mean ~ 151 µv) in RG indicating changes in cortical-excitability, as compared to CG (mean ~ 15 µv). The intergroup comparison, however, was not statistically significant (*p* = 0.65) between both groups (Table 2).

#### Inter-hemispheric differences and asymmetries

The effect of robotic-exoskeleton training on cortical excitability was assessed within both the hemispheres. RG showed statistically significant differences between ipsilesional and contralesional-sides as one factor and time points- pre and post-therapy as another factor on RMT (*p* = 0.049, F = 4.08), evidencing the dependence of time and hemisphere sides on each other. However, CG did not show any statistical differences (*p* = 0.06, F = 3.68).

RG also evidenced a statistically significant reduction in interhemispheric-RMT asymmetry as measured by the ratio of RMT for two hemispheres (RMT_asymm_ = RMT Ipsilesional / RMT contralesional) from pre-to-post-therapy (Table 2). RG showed a decrease in RMT_asymm_ from 1.43 ± 0.21 to 1.25 ± 0.31 (mean decrease of 0.18, *p* = 0.012), whereas, CG showed a decrease from 1.33 ± 0.30 to 1.30 ± 0.28 (mean decrease of 0.03, p = 0.59), indicating a trend of normalization of RMT-asymmetry (RMT_asymm_ should decrease as ipsilesional RMT should be decreased from pre to post) over the course of intervention in RG. RG also manifested statistically significant (p = 0.028) changes in intergroup comparison over CG (Table 2). The relative change in interhemispheric-RMT asymmetry-ratio (∆RMT_asymm-ratio_ = (Post RMT_asymm_—Pre RMT_asymm_) / Pre RMT_asymm_) changed with RG having a mean increase of 0.12 ± 0.14 and in CG a mean increase of 0.011 ± 0.1 (p = 0.028), indicating the extent of normalization of RMT-asymmetry over the duration of intervention in RG as compared to CG (Table 2).

### Relationship between TMS neurophysiological-measures and clinical-outcome

**Fig. 3:**
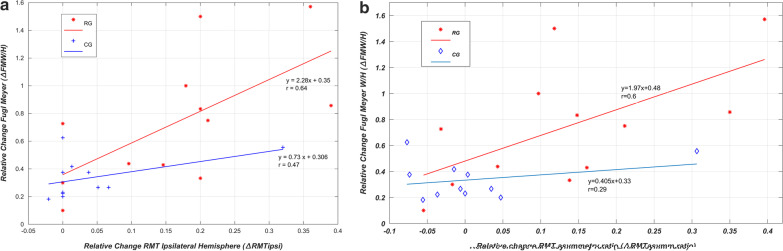
**a** Scatter-plot showing the relationship between improvements in RMT in the ipsilesional-hemisphere and improvements in functional performance of the distal-component pre-to-post-therapy for individual patient’s data. Greater decreases in motor-threshold tend to show greater increases in FMW/H. Red Line (RG) and the blue line (CG) represents a linear-trend in improvement in distal motor-outcome (∆FMW/H) score as a function of change in the ipsilesional-hemisphere (∆RMTipsi) in RG and CG pre-to-post-therapy in which RG shows a significant correlation (r = 0.64, F = 7.24, *p* = 0.022), **b** Scatter-plot showing the relationship between change in RMT asymmetry-ratio (ipsilesional/contralesional) pre-to-post-therapy and functional performance of distal-component for individual patient’s data. Greater decreases in motor-threshold tend to show greater increases in FMW/H. The red line (RG) and the blue line (CG) represents a linear-trend in improvement in distal motor-outcome (∆FMW/H) score as a function of change in RMT-ratio (∆RMTasymm-ratio) in RG and CG pre-to-post-therapy in which RG shows a significant correlation (r = 0.6, F = 5.77, *p* = 0.03)

The recovery parameters from TMS measures denoting the change from pre-to-post-therapy were observed to be correlated with the relative change/improvement in distal motor-outcome (∆FMW/H). The first parameter, the relative change in RMT in the ipsilesional-hemisphere (∆RMT_ipsi_ = (Pre RMT_ipsi_–Post RMT_ipsi_) / Pre RMT_ipsi_) was significantly (p = 0.0235) different for both the groups with a mean increase of 0.16 ± 0.12 in RG and 0.04 ± 0.09 in CG. The linear regression analysis indicated that ∆RMT_ipsi_ (as predictive/independent-variable) is moderately correlated with ∆FMW/H (as dependent-variable) and could predict ∆FMW/H in RG (r = 0.64, F = 7.24, *p* = 0.022) (Fig. 3a). This correlation was low in CG (r = 0.47, F = 2.62, p = 0.13) (Table 2, Fig. 3a). The relationship between ∆RMT_ipsi_ and ∆FMW/H for both groups is shown in the scatter-plot in Fig. 3a. The distal functional outcome ∆FMW/H also showed significantly (p = 0.012) different results for both groups with a mean increase of 0.73 ± 0.45 in RG and 0.33 ± 0.14 in CG.

The second parameter, the relative change in RMT-ratio (∆RMT_asymm-ratio_) was significantly (*p* = 0.028) different for both the groups. Similar to above, ∆RMT_asymm-ratio_ (as predictive/independent-variable) was observed to be moderately correlated with ∆FMW/H (as dependent-variable) (r = 0.6, F = 5.77, *p* = 0.03) (Fig. 3b), indicating that tendency towards the extent of normalization RG could be correlated and used further for predictive analysis of the clinical-outcomes in RG. This correlation was low in CG (r = 0.29, F = 0.83, *p* = 0.38) (Fig. 3b). The relationship between ∆RMT_asymm-ratio_ and ∆FMW/H in both groups is shown in the scatter-plot in Fig. 3b.

## Discussion

The study demonstrated improvement in both clinical and neurophysiological changes in patients with stroke in response to the robotic-exoskeleton [28] training compared to the conventional rehabilitation. The clinical scales showed an improvement in both RG and CG, however, increased cortical-excitability in the ipsilesional-hemisphere was shown only in RG with the appearance of MEPs in the ipsilesional-hemisphere post-therapy in patients. The improvement in RMT in ipsilesional-hemisphere showed a trend of normalization over the intervention and was also correlated with sensorimotor-functional improvement.

### Comparison of Clinical-scales of Robotic-therapy group with control-group

The robotic therapy was effective in releasing spasticity at the wrist joint with ~ 26% (*p* = 0.03) improvement over only ~ 14% in CG. The regain in normal muscle tone is considered as a predictor of recovery or the first step in recovery [45] followed by an increase in muscle strength and improvement in functional movements. Both groups showed significant improvement of AROM, RG showed significantly (*p* = 0.02) higher improvement of 130% over 47% in CG. (Table 2).

FMU/L, stroke-specific scale, is the most reliable measure of sensorimotor functionalities of the whole-arm [46]. RG established significantly higher improvement of ~ 40% over ~ 21% in CG (*p* = 0.039). For the FMW/H distal-component, both groups showed significant improvement, where RG showed significantly higher improvement of ~ 72% compared to only 32% in CG (p = 0.012) possibly because of intensive and repetitive training of wrist and MCP joints. However, RG did not show significant improvement in FMS/E, as expected as the intervention was not focused on the proximal component of the upper-limb. With contemporary studies showing improvements even in the proximal component in response to distal training, our study too reflected change in proximal joint (FMS/E) post-therapy, probably because of the compensatory muscle activities from the proximal-joints [12, 47].

The improvement in sensorimotor ability and functionality, evidenced by an increase in the FM, might pertain to diverse attributes of the device. The visual and proprioceptive feedback of performance in every trial encouraged and engaged the patient for the whole duration of the therapy-session. One aspect of the design that led to a substantial decrease in the spasticity is a maximum extension of fingers for a maximum stretch at the baseline position that is there at the end of every ten seconds trial repeating for 45 min of the therapy duration. The maximization of the voluntary muscle activity attempting for wrist extension in every ten-second trial repetitively could have led to an increase in passive and active range of motion attributing to increased mobility. Another facet contributing to the continuity and patient’s adherence during the therapy-session was the easy donning and doffing during the therapy-session leading to reposition of the hand multiple times required because of the instability in the position of hand during the therapy-session due to spasticity, a common presentation and rarely discussed obstacle in the rehabilitation of patients with spasticity. The improved spasticity and active range of motion, stability of wrist extension, and the achievable neutral position of the wrist over the duration of intervention, in summation, might have contributed to the improvement in the observed FMW/H scores. It is also worth noting that the patient cohort belonged to the sub-acute (n = 2) and only early chronicity of maximum 2 years (3 months to 2 years chronic stroke), which might be one of the underlying reasons triggering the neuroplasticity leading to an increase in FM.

Authors believe that a combination of multiple strategies used in the proposed robotic intervention could have possibly encouraged the clinically relevant neuroplasticity. The approach which would have been the key aspect of robotic-therapy over conventional therapy would be to use the movement goal that is specific, measurable, achievable, repetitive and timed [29]. It was also reinforced with maximizing voluntary residual muscle activity (using EMG thresholds based on individual volitional effort) with real-time adaptive visual extrinsic performance biofeedback and intrinsic proprioceptive feedback for sensorimotor integration in every cycle of the movement as synergy based training approach for maximizing brain reorganization [30, 31].

As shown in the studies by Gladstone et al. and Shin et al. [48, 49], a value of 6.6 (FMU/L) reflects the potential Minimally Clinically Important Difference (MCID). In our study, FMU/L (14 on a scale of 66) was found to be higher than the MCID values for all 12 patients in RG and 7/11 patients in CG. The Hand Mentor Pro reported improvement in 99 patients with stroke with FMW/H being 5.6, FMU/L 10.33 in combination with a home exercise program (which alone reported FMW/H 4.9 and FMU/L 9.3) [50]. The HWARD [47] also showed an improvement in FMW/H (~ 4) with sensorimotor cortex laterality index representing a shift in interhemispheric balance over time from the contralateral to the ipsilateral side and also suggested the use of synchronizing both wrist and MCP-joint movement in grasp and release.

With Constraint-Induced Movement therapy (CIMT), the reported gain is FMU/L ~ 13 and BI ~ 13.5, post three weeks therapy [51]. Moreover, a systematic review and meta-analysis have shown an improvement in FMU/L scores Action Research Arm Test (ARAT) scores with improved control of hand and arm placement as well as improved strength compared to standard therapy post-CIMT in the subacute and chronic stroke population [52]. Few studies have also shown significantly increased motor map area via TMS post-CIMT [3, 53]. Sawaki et al., showed an increase in the TMS motor map area (of EDC muscle in ipsilesional hemisphere in few patients) and clinically relevant improvement in arm motor function that persisted for at least 4 months, however, other TMS parameters like Resting Motor Threshold, Active Motor Threshold, Center of Gravity and the silent period did not change over time [54]. The use of biofeedback has been another widely explored area, where Doan-Aslan et al. and Zheng et al. has demonstrated an increase in AROM, BI, and FMU/L in patients with the stroke while using EMG Biofeedback compared to the conventional therapy [55, 56]. In our study, an improvement in FMW/H by ~ 3 was observed in the CG, which is consistent with the literature. Krishnan et al. and Calabrò et al. have attempted to evaluate the effect of active robotic training on changes in cortical-excitability, using commercially available devices, such as Lokomat robot (lower-limb) [57] and ARMEO (upper-limb) [58]. With very sparse literature exploring cortical excitability changes in lower-limb [59] and upper-limb [60], virtual mirror task with feedback demonstrated increased MEP by up to 46.3% (95% CI: 30.4 ~ 80.0) compared with the real mirror task [60].

For the BI, both groups showed similar (~ 20%) improvement (*p* = 0.82). Both groups showed significant improvement for BS as well, however, RG showed ~ 32% improvements compared to only ~ 20% in CG (Table 2). In the case of the Barthel Index and Brunnstrom Stage, both RG and CG showed a similar improvement as the rehabilitation regimen in the CG group incorporated clinical rehabilitation with a primary focus on the upper extremity deficits with the therapist focusing on the distal limb and overall recovery along with customizing the patient’s goals directly and training compensatory and functional movement strategies that consequently resulted in equal gains in independence and patients’ goals as in RG. Also, in the future, substantial consideration can be given to Barthel Index scores by introducing kinematic analysis of speed, accuracy, and precision of movement and BI-based patient perception scales like self-perceived difficulty scale and ability scale for better quantitative measurement.

### Comparison of Cortical-excitability of Robotic-therapy with the control-group

For healthy subjects, MEP ranges 186.4 ± 88 µv at 55 ± 10 stimulation intensity at 100% RMT [33]. In patients with no MEP recorded due to low cortical excitability in stroke, RMT is taken as a value of 100, as suggested in the literature [43, 44]. Though a subset of patients with stroke with affected corticospinal tract integrity does not demonstrate MEP with the highest stimulation intensity, taking RMT as 100% could affect the decrease in RMT post-therapy in the RG group. However, critical studies like Hendrics et al. and Jong et al. have established MEP as a sensitive and valid prognostic marker of motor recovery after stroke [61–63].

#### Ipsilesional and Contralesional-hemisphere changes

With the decrease in RMT, RG showed ~ 16% improvement as compared to only ~ 4% improvement in CG (p = 0.037). Interestingly, RG showed a significantly (*p* = 0.048) higher increase in MEP amplitude post-therapy with an increase of ~ 140% (mean = 54.9 µv), whereas CG showed no such improvement. Cortical-excitability measures are used as an objective investigative tool to measure the treatment responsiveness and prognostication as they provide insights into membrane-excitability of neurons, conduction, and functional integrity of the corticospinal tract and neuromuscular-junctions [64]. A decrease in RMT and an increase in MEP amplitude in the ipsilesional-hemisphere demonstrated in the RG and not in CG, might be related to the increase in cortical excitability [65]. It might be interpreted that the recovery of motor function could most likely be a consequence of plastic reorganization and use-dependent plasticity [65]. Cortical-excitability and corticospinal tract integrity have also been shown to be correlated with functional recovery potential in patients with chronic stroke [39]. The exoskeleton training appears to be beneficial in activating the ipsilesional-hemisphere for our patient cohort (chronicity 13.8 ± 9.1 months). Activation of ipsilesional-hemisphere could indicate either vicariation of the loss of neural circuits or unmasking of preexisting synapses or recruitment of perilesional areas in ipsilesional-hemisphere or exploitation of the preserved functional recovery reservoir in ipsilesional-hemisphere [43, 66–68].

In the contralesional-hemisphere, MEP amplitude showed a considerable decrease in both groups, though not significant, RG evidencing a decrease of ~ 30% (mean = 151.03 µv) and CG a decrease of only ~ 7% (mean = 14.8 µv) with no inter-group significant differences (*p* = 0.65) (Table 2). A ~ 30% decrease in MEP amplitude in contralesional-hemisphere over the duration of intervention might represent a decrease in cortical-excitability [66, 67], however, is difficult to comment on it at this stage due to the small sample size and needs to be further evaluated in a larger cohort.

The potential clinical effectiveness harnessed by the neuro-rehabilitation robots has also been shown by few studies in terms of subjective clinical scales or questionnaires or EMG parameters which might not be sufficient to assess cortical reorganization [7, 10, 12–15, 69–73]. However, the mechanism of entrainment of neuroplasticity followed by a stroke that favors motor learning and functional recovery is still unclear [74]. Despite recognizing that the corticospinal tract plays a critical role in recovery potential, cortical reorganization, functional improvement in stroke, and as well as to better track clinical progression; the changes in these measures evaluating effects due to intervention are usually limited to the studies involving brain stimulation protocols. Examples are repetitive TMS, Transcranial Direct Current Stimulation (tDCS) [75, 76], etc. or in a combination of brain-stimulation with other neuro-rehabilitation strategies like CIMT [77] or mirror-therapy [78] or training [59, 60]. Hence, only limited studies are available assessing for these measures unveiling objective changes using robotic therapy as a rehabilitation intervention [59, 60, 75–81].

Though the study using the device HWARD provided seminal evidence of reorganization of brain (via fMRI), as well as motor function in response to the robotic therapy, no direct comparison can be made with our study as different modalities—TMS and fMRI were used to measure different neurophysiological aspects [47]. Juan et al. correlated results by these modalities and presented that larger fMRI activation likelihood and motor cortical excitability in the ipsilesional primary motor area were related to improved motor performance [82].

#### Specific five-patients in RG

A very critical outcome of this therapy was that in RG, MEP was evoked in ipsilesional-hemisphere only for 4/12 patients at the pre-therapy measurements; whereas, MEP was later evoked for 9/12 patients post robotic therapy. However, in CG, MEP was evoked only for 5/11 patients and was later evoked for 6/11 patients at post-therapy. Considering these five specific patients in RG who did not evoke MEP at pre-therapy (0 µv) and later evoked MEP (mean = 136.6 ± 38.48 µv), with a decrease of stimulation intensity in ipsilesional-hemisphere by almost 27% and substantial improvement in the value of clinical-scales (FMW/H: 7.8 ± 2.38, BI: 22 ± 11.72, AROM: 22^0^ ± 2.73^0^). These changes were relatively much higher than the changes in patients who already had MEP evoked at pre-therapy measures. The appearance of MEP in five patients after 4 weeks of robotic intervention is a crucial outcome and represents that the robotic therapy might have the potential of facilitating clinically relevant reorganization of brain-based on use-dependent plasticity. The observed increase in cortical-excitability and normalization of TMS neurophysiological markers on the ipsilesional-side are also accompanied by recovery of hand-function, as observed by sensorimotor and functional recovery (by clinical-scales FMW/H, BI & AROM).

#### Inter-hemispheric differences and asymmetries

The diaschisis between ipsilesional-areas and intact neuronal-networks of contralesional-areas may disturb the cortical-excitability and connectivity-patterns of connected, remote, or primary-motor areas of contralesional-hemisphere (via transcallosal-fibers). The effect of robotic-exoskeleton training on cortical-excitability of both hemispheres might be attributed to remodeling of the bilateral primary-motor areas in RG which is not shown in CG.

For cortical excitability to be increased in ipsilesional-hemisphere for patients with stroke, the ipsilesional-RMT should be decreased from pre-to-post-therapy and hence, RMT_asymm_ (RMT ipsilesional/RMT contralesional) should decrease to approach normalization [43]. When TMS-neurophysiological parameters were expressed in terms of the interhemispheric-asymmetry ratio RMT_asymm_, significant differences (p = 0.028) were observed between the groups post-therapy. It might be a representative of trend towards the normalization of asymmetry of TMS-measures in RG in response to exoskeleton-training. Normalization might indicate the recruitment of peri-lesional areas in the ipsilesional-hemisphere or exploitation of the preserved functional-recovery reservoir in the ipsilesional-hemisphere [43, 66–68].

### TMS neurophysiological improvement correlating the motor-outcome of both groups

The amount of change in TMS neurophysiological-measures of corticomotor-pathways (∆RMT_ipsi_ and ∆RMT_asymm-ratio_) were found to be associated with the amount of improvement observed in the functional motor-outcome during the rehabilitation of the distal-part of upper-limb (∆FMW/H) (Fig. 3). The improvement (decrease) in RMT, could be associated with recovery of motor function as suggested by [39]. This might be most likely due to increased cortical-excitability of preserved motor-pathways as shown in earlier studies in sub-acute and chronic stroke, demonstrating the correlation of improvement in TMS neurophysiological-measures (improvement in RMT and normalization) with functional improvement [43, 83, 84]. It is also worth mentioning that these neurophysiological measures were obtained specifically from the cortical representation of EDC muscle, a clinically affected muscle, with a specific function that was involved in training with the robotic exoskeleton, whereas most clinical measures do not necessarily require a particular muscle group and measures motor function in a broader sense.

Also, these neurophysiological-parameters individually establishes as a good correlation of functional rehabilitation-outcome of hand (∆FMW/H) in RG, indicating that changes in cortical-excitability of ipsilesional-hemisphere might be used to correlate with the clinical-outcome, emerging as recovery parameters to be considered, however, its predictive evidence has to be evaluated in the future with a larger data-samples. This could be the plasticity markers of the responsiveness of chronic post-stroke patients [58].

Since RG and CG had very similar lesion locations and size with all patients having their motor paths affected, an increase in cortical excitability can thus be attributed to the different interventions received by the groups. There was a limited number of cases in subgroups, i.e. only 2/12 patients from the RG and 2/11 patients from the CG belonged to the subacute stage (3–6 months), the majority of patients are chronic (< 2 years). Also, the CG included 5 subcortical and 6 cortical stroke and RG included 6 subcortical and 6 cortical stroke. Considering the threshold for recovery as MCID for FMU/L 6.6 [48, 49], out of 11 patients in CG, a total of 7 patients (5 sub cortical and 2 cortical) exceeded this threshold and all twelve patients in RG exceeded this threshold. Any conclusion on the trend for sub-acute or chronic stroke and the responders or the non-responders to the intervention would be highly presumptive at this stage due of the small sample size.

### Limitations and future work

Even though the data are promising, the study had few limitations such as small sample size and lack of an activity level measurement like Wolf Motor Function test and Action Research Arm Test, no midterm clinical assessment, and long term follow-up of patients. As most of the patients at our quaternary hospital came from far places across India, it was not possible to follow up with them once they have left the city. Another limitation was therapist performing both sets of interventions could not be blinded to the group allocation. There are several ways the study could be improved. The sample size could be increased and patient groups could be further subdivided into sub-acute and chronic stages to evaluate any difference in rehabilitation outcomes*,* with mid-term clinical assessment and long-term follow-up with the double-blinded protocol. Different distal goal-directed and translation to home measures could be included like WMFT or ARAT, Functional Independence Measure, Canadian Occupational Performance Measure or Motor Activity Log, nine-hole pegboard, stroke Impact scale and Interhemispheric Inhibition measures using TMS, etc. The device currently is in the prototype stage with clinical validation, thus the BIOPAC EMG system was used in data acquisition for research and validation. In the future, this will be easily replaced by an MYOWARE or an in-house built EMG amplifier. The device will have an LCD touch screen for settings and feedback. These features will make the system more aesthetic, compact, and accessible. Once the device is further optimized in terms of weight, aesthetics, and compactness, it can be deployed for home-based rehabilitation in the future. Also, with a minor modification, the device can synchronize wrist extension with finger extension which can be further explored for outcome in patients with stroke.

## Conclusion

Robotic intervention group showed improvement in both the clinical scales and the neurophysiological parameters in the ipsilesional hemisphere as compared to conventional physiotherapy. This improvement could only be a consequence of plastic reorganization and use-dependent plasticity; as therapeutic intervention was the only difference among both the cohorts. The goal-oriented robotic-therapy using exoskeleton might have future implications in facilitating the recovery of stroke with this neuro- rehabilitation device. The outcomes provided critical evidence to plan a multi-centric trial with a large cohort size in the future to systematically investigate the potential of the exoskeleton device in clinical practice.

## Supplementary Information

Additional file 1: **Figure S1.** Exoskeleton device in baseline position (top), Exoskeleton device in final position (bottom). Details of ‘Conventional therapy-sessions’. **Figure S2.** Plot showing Fugl-Meyer scores of RG (n=12) and CG (n=11) pre and post-therapy.

## Data Availability

The datasets used and/or analyzed in the current study are available from the corresponding author on reasonable request.
