# Supplementary material for: Evidence of neuroplasticity with robotic hand exoskeleton for post-stroke rehabilitation: a randomized controlled trial
Source: J Neuroeng Rehabil. 2021 May 6;18:76. doi: 10.1186/s12984-021-00867-7 (PMC8101163; doi:10.1186/s12984-021-00867-7)
Supplement: Supplementary file 1 — Additional file 1: Figure S1. Exoskeleton device in baseline position (top), Exoskeleton device in final position (bottom). Details of ‘Conventional therapy-sessions’. Figure S2. Plot showing Fugl-Meyer scores of RG (n=12) and CG (n=11) pre and post-therapy. [file 12984_2021_867_MOESM1_ESM.docx]

**EVIDENCE OF NEUROPLASTICITY WITH ROBOTIC HAND EXOSKELETON STUDY**

**FOR POST-STROKE REHABILITATION: A RANDOMIZED CONTROLLED TRIAL**

**Supplementary material**

**
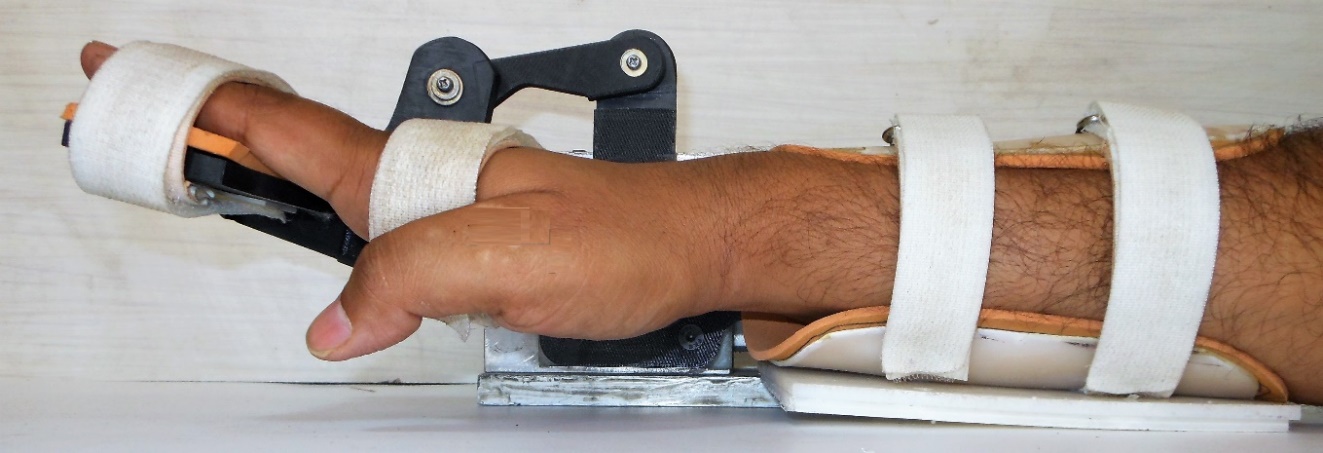

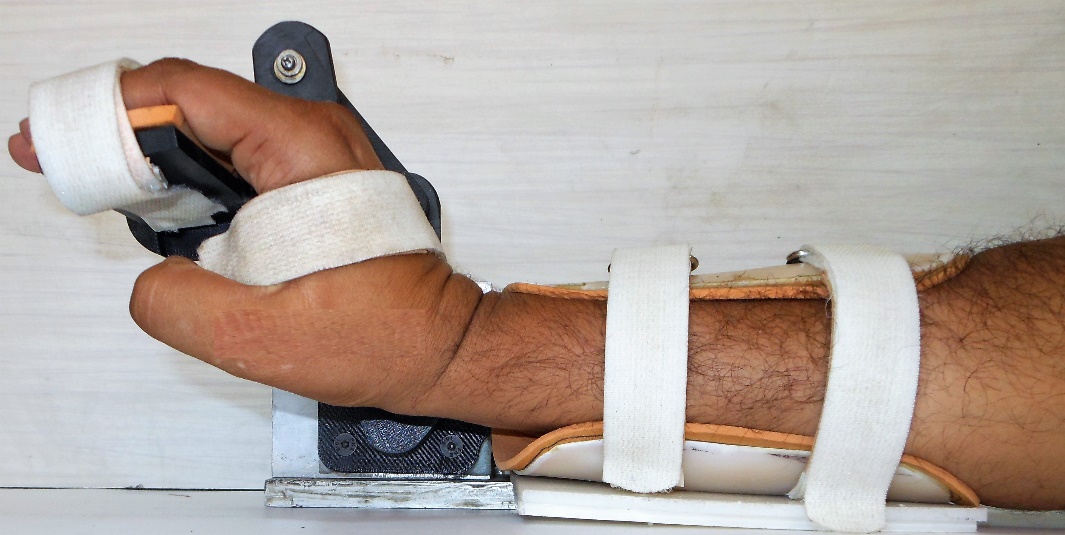
**

**Supplementary Figure 1: Exoskeleton device in baseline position (top), Exoskeleton device in final position (bottom)**

(This figure reprinted by permission from IEEE Trans Neural Syst Rehabil Eng, from an article by Singh N et al.) Singh N, Saini M, Anand S, Kumar N, Srivastava MVP, Mehndiratta A. Robotic Exoskeleton for Wrist and Fingers Joint in Post-Stroke Neuro-Rehabilitation for Low-Resource Settings. IEEE Trans Neural Syst Rehabil Eng [Internet]. 2019 [cited 2019 Sep 27];1–1. Available from: https://ieeexplore.ieee.org/document/8846101/

**Details of ‘Conventional therapy-sessions’:**

- Upper extremity paresis is an impairment experienced by patients with stroke in our cohort. Moreover, as established in previous studies that patient perception of recovery from upper extremity paresis is considerably influenced by the degree of recovery in paretic hand and fingers therefore, motor relearning exercises focused on the wrist and hand gross and fine movement deficits, limiting patients active functional participation in both basic and instrumental activities of daily living were implemented.
- Training session for the control group was conducted for 45 min for 5 days a week for 4 weeks, the same as for the intervention group. The type of activity, intensity, and frequency was based on the baseline clinical presentation of the individual patient as reflected by clinical scales (MAS, FMA, BI, Brunnstorm scale, and Range of motion).
- Tasks specific to upper extremity included:
- Passive stretching of long wrist flexors with the hold of 30 sec followed by relaxation
- Fist making in prone and thrust release-10 reps (3sets)
- Fist making in mid prone position -10 reps (3sets)
- Ball squeezing and release-10 reps
- Lock and key movements -10 reps
- Muscle facilitation techniques like stroking, brushing, icing - 3-5 strokes twice in a day
- Task-oriented training (TOT); reaching, grasping a tool, lifting a glass of water, pen holding, bottle opening.
- The difficulty of the task was gradually progressed depending on improved performance of upper extremity activities like reaching, grasping, lifting, steady holding at multiple task-related joint angles which requires an optimal range of motion of the shoulder, elbow, wrist, and hand; smooth inter-joint coordination and the optimal speed of doing a task as compared with the individual patient unaffected extremity.
- The task was made challenging by adding gradations like:
- Gross to fine movements
- Static to dynamic positioning
- Single to dual activity
- Non weighted to the incorporation of weights
- Indoor to the outdoor environment
- In case the patient experiences any kind of pain or fatigue in wrist/hand, proximal joints like shoulder/elbow; balance or coordination difficulties while doing a particular task, the task was fragmented in small segments for the ease of practice and was gradually built up from there for achieving the required functional goal.

**
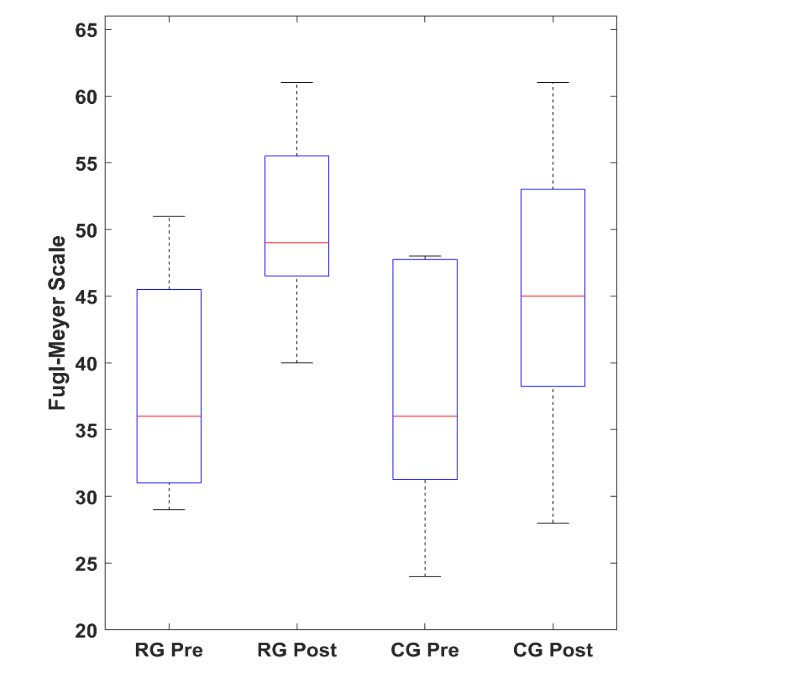
**

**Supplementary Figure 2: Plot showing Fugl-Meyer scores of RG (n=12) and CG (n=11) pre and post-therapy**
